# What to do when advanced thyroid cancer invades the carotid artery? Therapeutic challenge

**DOI:** 10.1590/1677-5449.202102201

**Published:** 2022-09-19

**Authors:** Vinicius Tadeu Ramos da Silva Grillo, Rodrigo Gibin Jaldin, Matheus Bertanha, Marcone Lima Sobreira, Carlos Segundo Paiva Soares, Paula Angeleli Bueno de Camargo

**Affiliations:** 1 Universidade Estadual “Júlio de Mesquita Filho” – Unesp, Faculdade de Medicina de Botucatu, Botucatu, SP, Brasil.

**Keywords:** thyroid neoplasms, carotid artery injuries, vascular system injuries, vascular surgical procedures

## Abstract

Of all thyroid cancers, anaplastic thyroid carcinoma (ATC) has the lowest incidence and worst prognosis. In this report, we describe a 64-year-old female patient who underwent total thyroidectomy and level VI neck dissection for papillary thyroid carcinoma. During follow-up, she showed signs of regional recurrence and underwent extended neck dissection and cervical esophagectomy. Intraoperatively, there was no cleavage plane between the tumor and the common carotid artery (CCA), so a carotid shunt was implanted and en bloc resection, including the affected CCA and esophagus segments was performed followed by vascular bypass with interposition of a great saphenous vein graft. A pathology review found evidence of anaplastic carcinoma. The patient underwent adjuvant treatment and has no signs of locoregional recurrence. Presented with the possibility of carrying out curative surgery with en bloc resection, the vascular surgeon must be prepared for the surgical options.

## INTRODUCTION

Anaplastic thyroid carcinoma (ATC) originates from the follicular cells of the thyroid. Of all the histological subtypes of thyroid cancer, ATC has the lowest incidence and worst prognosis. There are few data on its incidence in Brazil, but in the United States it accounts for about 1.7% of thyroid cancers and has overall 1-year survival of 20%.^1^

Its sudden appearance and rapid growth, with invasion of structures that can make initial surgical treatment unfeasible, make early diagnosis and multidisciplinary management with a team of surgeons, endocrinologists, radiotherapists, and clinical oncologists essential.^2^ When ATC has invaded the common carotid artery, prognosis is poor and the only treatment with curative potential is surgical en bloc removal of the tumor with resection of the carotid.^3^

This study was duly assessed and approved by the Research Ethics Committee (CAAE 52619521.2.0000.5411, decision number 5.086.455).

## PART I – CLINICAL SITUATIONS

**Figure 1:**
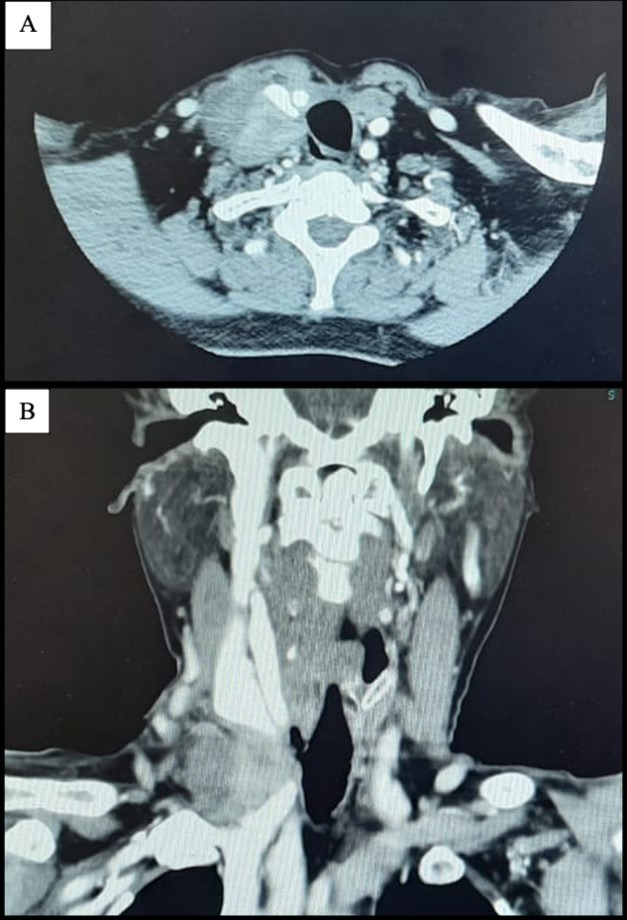
Computed tomography showing: (A) tumor in intimate contact with the posterolateral portion of the right internal jugular vein, esophageal wall, and right sternocleidomastoid muscle (axial slice); and (B) the same tumor and its relationship to the convergence of the right internal jugular vein and the right subclavian vein (coronal slice).

A 64-year-old, female, white patient with hypertension and asthma, but independent for basic activities of daily living (BADL), presented complaining of productive cough, fever, and dyspnea and was diagnosed with community acquired pneumonia and treated with antibiotic therapy. Since the symptoms were refractory to conventional treatment, chest tomography was ordered, showing bronchi with thickened walls and fine linear atelectasis, suggesting inflammatory bronchopathy. An additional finding was observation of increased dimensions of the right lobe of the thyroid, which projected inferiorly to the anterior mediastinum, displacing the trachea to the left (Figure 1). Investigation was continued with thyroid ultrasonography, which showed that the left lobe of the thyroid was unchanged (measuring 4.03 × 1.5 × 12 cm, with a volume of 4.1 cm^3^) and the right lobe of the thyroid was enlarged (measuring 6.2 × 3.2 × 3.1 cm, with a volume of 30.5 cm^3^), occupied by a hypoechogenic nodule, with heterogeneous echotexture, measuring 5.5 × 3.1 × 2.8 cm. Laboratory tests results included free T4 of 0.42 ng/dL, thyroid stimulating hormone (TSH) of 1.69 mUI/L, and a thyroid peroxidase (anti-TPO) result below 3 IU/mL.

A fine-needle aspiration biopsy (FNAB) of the thyroid nodule was performed. Cytology findings were as follows: highly cellular material with clusters of overlapping cells, rudimentary papilla, enlarged nuclei, pale chromatin, and intranuclear cytoplasmic pseudoinclusions, amongst hyaline material, multinucleated giant cells, and red blood cells. These findings are compatible with papillary thyroid carcinoma (Bethesda VI).^4^

The patient underwent total thyroidectomy and level VI neck dissection,^5^ yielding anatomopathological evidence of papillary thyroid carcinoma, classic variant, measuring 5 × 4 cm in the right lobe of the thyroid and with foci of involvement in the left lobe and isthmus, in addition to a macroscopic extrathyroid extension, angiolymphatic and perineural invasion, compromised radial surgical margins, and metastasis in two of the five resected lymph nodes (anatomopathological staging: pT3bpN1apMx).^6^ The patient was classified as at high risk of relapse according to the criteria in the American Thyroid Association guideline for differentiated thyroid cancer^2^ and treated with radioactive iodine therapy, at a dose of 150 mCi.

In outpatients follow-up, signs of regional relapse were detected 9 months later, manifest as lymphadenomegaly of around 6 cm involving cervical levels III, IV, and V and in intimate contact with the right internal jugular vein, wall of the esophagus, and right sternocleidomastoid muscle (RSM), incompatible with stimulated thyroglobulin (Tg) tests (TSH = 69 and Tg < 0.04). The patient was therefore scheduled for neck dissection at levels II to V, extended to cervical esophagectomy .

She was brought in for surgery, starting with a cervical hockey-stick incision from the tip of the right mastoid to the mid-low region of the neck. Dissection of the lymph node conglomeration (LNC) was begun at the lower internal jugular chain (level IV) and inferior portion of the posterior triangle of the neck (level VB). During this stage, it was found that there was no cleavage plane between the LNC and the right internal jugular vein (RIJV) and that this was insinuated close to the right subclavian vein (RSV). It was decided to perform ligature of the RIJV and a claviculectomy to improve visibility and access for dissection of the RSV. Clearance of levels II, III, and V was resumed. At level V, there was no visible cleavage plane between the right accessory nerve and the LNC, so the nerve was sectioned. At level II, ligature of the proximal portion of the RIJV was performed. During dissection for transition from level III to IV, it was observed that the LNC did not offer a cleavage plane to the right common carotid artery (RCCA), or the RSM, extending directly to level VI, and also did not offer a cleavage plane to the proximal portion of the cervical esophagus.^5^ It was decided to section the RSM at its proximal portion and proximal of its insertion into the sternoclavicular and resection a segment of around 3.5 cm from the posterolateral wall of the esophagus, which was promptly repaired by the gastrointestinal surgery team by esophagography in two planes and coverage with patch of the adjacent musculature, in addition to a Witzel jejunostomy. In view of the intraoperative finding of no cleavage plane between the LNC and the RCCA, the vascular surgery team’s opinion was requested.

If it was decided to proceed with resection en bloc of the common carotid, the options for vascular surgery tactics would be:

- Ligature of the common carotid artery; or
- Temporary vascular shunt to enable definitive vascular reconstruction:
  - Vascular reconstruction with polytetrafluoroethylene (PTFE) or dacron prosthetic graft;
  - Vascular reconstruction with autologous vein; or
  - Vascular reconstruction with autologous artery.

## PART II – WHAT WAS DONE

Faced with the absence of a cleavage plane between the LNC and the RCCA, the team proceeded to dissection and isolation of the RCCA, from the base of the neck to the carotid bifurcation, and of the external and internal carotid arteries from the carotid bifurcation to the point at which they cross the posterior digastric muscle belly. They were then repaired with cardiac tape.

The option chosen was a temporary vascular shunt and definitive vascular reconstruction with an autologous vein graft, so a distal segment of the great saphenous vein (GSV) was dissected and harvested.

**Figure 2:**
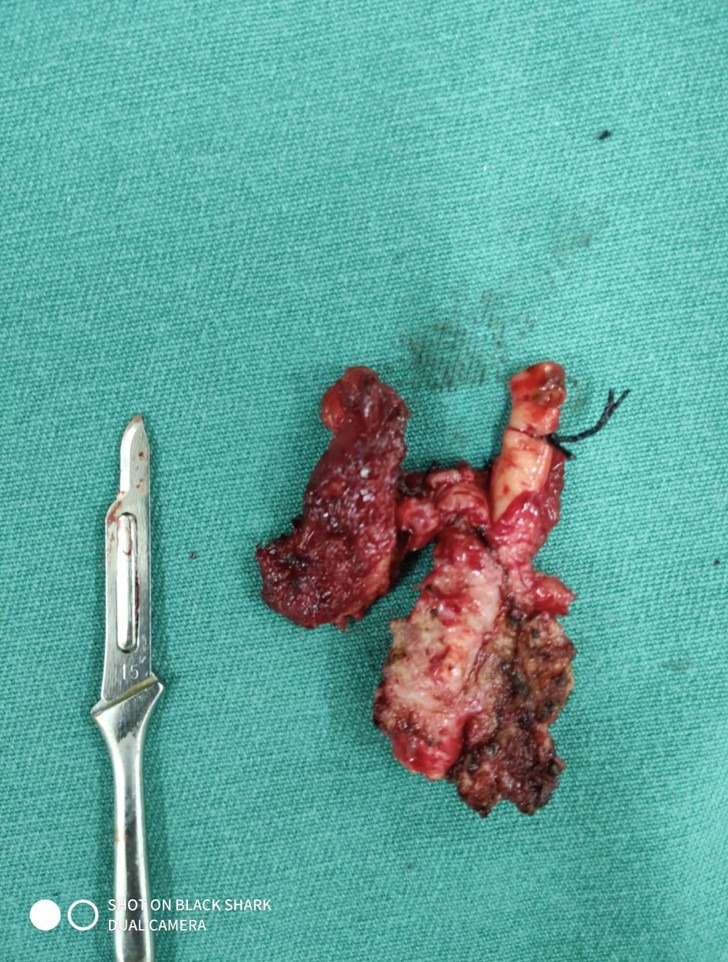
Resected surgical specimen showing the lymph node conglomeration and its relationship to the segment of the right common carotid artery. On the left: number 15 scalpel blade to provide scale.

**Figure 3:**
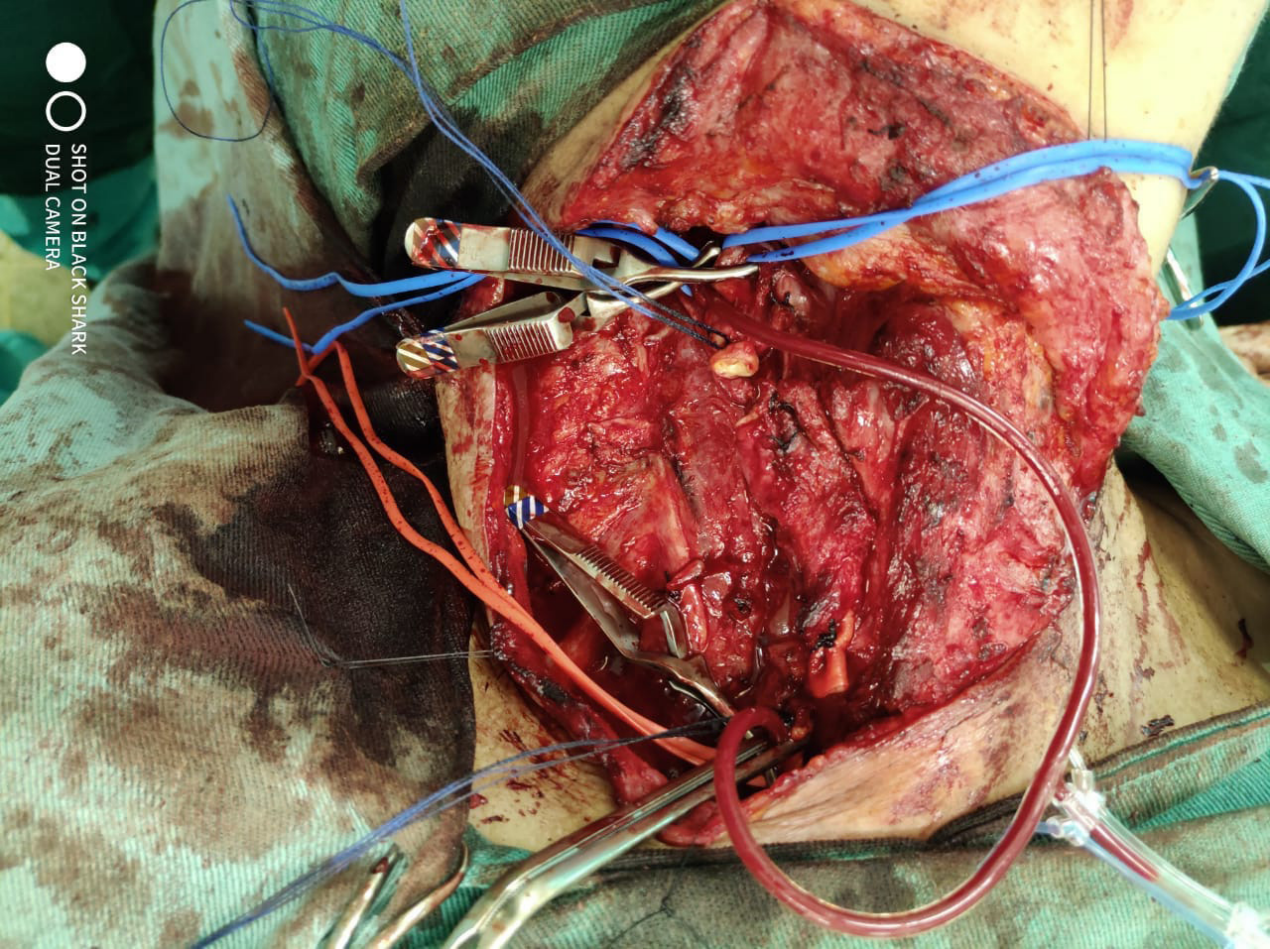
Intraoperative photograph during surgery, showing position and function of the vascular shunt after removal of the lymph node conglomeration. The red vascular ties repair the common carotid artery and the blue ties repair the internal and external carotid arteries.

After administration of 5,000 IU of intravenous unfractionated heparin, the RCCA was clamped proximally and distally at the right internal carotid artery (RICA) and right external carotid artery (RECA). A transverse arteriotomy of the RICA and RCCA was performed at a safe distance from the oncological resection, followed by implantation of the Pruitt F3^®^ temporary carotid shunt (LeMaitre Vascular, Burlington, United States). After achieving vascular control, it was possible to resect the LNC together with the involved segments of the RCCA and esophagus (Figures 2 and 3).

**Figure 4:**
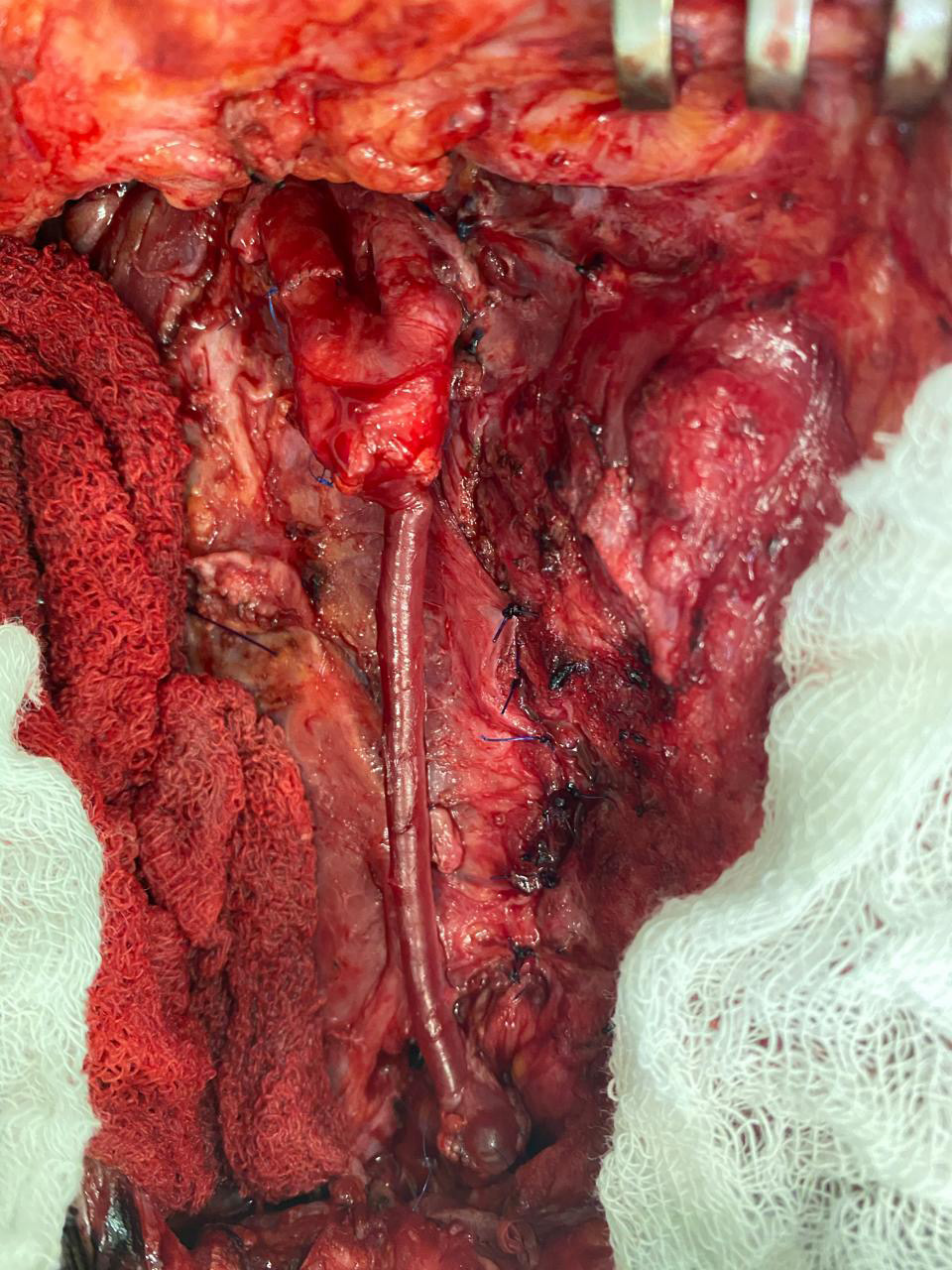
Intraoperative photograph of the right carotid vascular bypass by interposition of a great saphenous vein graft. Note the arteriorrhaphy of the internal carotid at the site where the distal part of the vascular shunt had been.

The GSV graft was interposed with end-to-end anastomoses, proximally to the RCCA and distally to the carotid bifurcation, using polypropylene 6-0. At the end of the procedure, the temporary carotid shunt was removed and palpable pulsation was observed both before and after the anastomoses (Figure 4).

Review of the anatomopathological slide from the first surgery found anaplastic thyroid carcinoma, rhabdoid variant, accounting for 10% of the tumor, evolved from classic variant papillary thyroid carcinoma, which accounted for 90% of the tumor, with extrathyroid extension, angiolymphatic and perineural invasion, and metastasis to two of five lymph nodes affected.

In turn, anatomopathological analysis of the specimen from neck dissection revealed metastasis of papillary thyroid carcinoma to three lymph nodes and of anaplastic thyroid carcinoma to three of the 16 lymph nodes analyzed.

Finally, the patient underwent adjuvant treatment with radiotherapy and chemotherapy. Currently, she has been in outpatients follow-up for about 7 months since the end of adjuvant treatment and has not shown any signs of locoregional relapse.

## DISCUSSION

Vascular invasion, vascular compression, or thrombosis are rare complications of differentiated thyroid cancer.^3^^,^^7^ The vascular structure most often involved in occlusion or invasion is the internal jugular vein, while the carotid artery is less commonly affected.^3^^,^^8^

The most important factor in any planned en bloc resection involving vascular reconstruction is assessment of the contralateral cerebral blood flow and intraoperative management of ischemia duration.^3^^,^^7^

There is near consensus among specialists that in cases of focal vascular invasion, the wall of the vessel can be resected after appropriate proximal and distal control, followed by reconstruction with a patch.^3^

In some patients with advanced thyroid cancer, low life expectancy, poor prognosis, difficult to control bleeding with risk of death, a thrombosed stump with risk of embolization during handling, and severe neurological injury with prior sequelae, resection of the carotid artery without vascular reconstruction is an option.^7^^-^9 However, a resection followed by reconstruction is superior to simple ligature because it avoids major postoperative neurological complications.^10^

Therefore, as in other situations, after taking the decision to perform vascular reconstruction, and when the carotid is involved but not occluded, a shunt is indispensable to enable safe reconstruction of the carotid artery.^3^^,^^7^^,^^8^^,^^11^

Reconstruction can be accomplished with an autologous graft, of which the great saphenous vein is the most often used, or using biomaterials such as PTFE. Although it offers mechanical resistance, safety, and durability, because PTFE is not adherent, it offers a relatively non-thrombogenic surface with an increased risk of infections.^10^

The risk factors for infection include handling the mucosa of the aerodigestive tract, presence of tracheostomy, and manipulation of previously irradiated tissues, in addition to occurrence of salivary fistulas.^7^

The best option for reconstruction is autologous vein graft, which offers the most durable patency and lower numbers of thrombotic and infectious complications.^3^^,^^7^

Vascular reconstruction procedures may be associated with neurological complications, but with lower incidence than after simple ligature.^9^ Nevertheless, the elevated risk of neurological damage or death and the low rates of cure intimidate many surgeons not to perform carotid artery resection.^10^

With regard to the vascular management chosen in the case reported, simple ligature of the RCCA was rejected because the patient was young, independent for BADL, and had a chance of cure. The temporary shunt was used because the decision to employ this surgical tactic had already been taken prior to the decision to perform en bloc exeresis of the tumor. With regard to vascular reconstruction, a prosthetic graft was rejected because of the heightened risk of infection due to the associated esophageal injury, so reconstruction was accomplished with autologous vein.

One limitation of this study is not having performed another imaging exam during postoperative follow-up to assess graft patency.

## CONCLUSIONS

Presented with the possibility of performing curative thyroid surgery with resection en bloc with the carotid, the vascular surgeon should be prepared for the surgical options in such a situation, of which a temporary shunt and vascular reconstruction with autologous vein are the most recommended.
