# Supplementary material for: What to do when advanced thyroid cancer invades the carotid artery? Therapeutic challenge
Source: J Vasc Bras. 2022 Sep 19;21:e20210220. doi: 10.1590/1677-5449.202102201 (PMC9499725; doi:10.1590/1677-5449.202102201)
Supplement: Translation

# Translation: O que fazer quando o câncer de tireoide avançado invade a artéria carótida? Desafio terapêutico

**DOI:** 10.1590/1677-5449.202102202

**Authors:** Vinicius Tadeu Ramos da Silva Grillo, Rodrigo Gibin Jaldin, Matheus Bertanha, Marcone Lima Sobreira, Carlos Segundo Paiva Soares, Paula Angeleli Bueno de Camargo

**Affiliations:** 1 Universidade Estadual “Júlio de Mesquita Filho” – Unesp, Faculdade de Medicina de Botucatu, Botucatu, SP, Brasil.

**Keywords:** neoplasias da glândula tireoide, lesões das artérias carótidas, lesões do sistema vascular, procedimentos cirúrgicos vasculares

## Abstract

O carcinoma anaplásico da tireoide corresponde ao câncer de menor incidência e de pior prognóstico. Neste relato, descrevemos o caso de uma paciente de 64 anos submetida a tireoidectomia total associada a esvaziamento cervical nível VI devido a carcinoma papilífero de tireoide. No seguimento, apresentou sinais de recidiva regional e foi submetida a esvaziamento cervical ampliado e esofagectomia cervical. No intraoperatório, observou-se ausência de plano de clivagem entre o tumor e a artéria carótida comum (ACC) e procedeu-se com implante de *shunt* carotídeo, resseção em bloco incluindo segmento da ACC e do esôfago acometidos e ponte vascular com interposição da veia safena magna. Foi evidenciado carcinoma anaplásico em revisão de lâmina do exame anatomopatológico. A paciente foi submetida ao tratamento adjuvante e não apresenta sinais de recidiva locorregional. Diante da possibilidade de proceder uma cirurgia curativa com ressecção em bloco, o cirurgião vascular deve estar apto para as opções cirúrgicas.

## INTRODUÇÃO

O carcinoma anaplásico da tireoide (CAT) origina-se das células foliculares da tireoide. Entre todos os subtipos histológicos de câncer desse órgão, o CAT corresponde ao de menor incidência e de pior prognóstico. Há poucos dados da sua incidência no Brasil, mas, nos Estados Unidos, ele corresponde a cerca de 1,7% dos cânceres de tireoide, sendo de 20% a sobrevida global em 1 ano^1^.

Em função do seu aparecimento repentino e crescimento rápido, com invasão de estruturas que podem inviabilizar o tratamento cirúrgico inicial, é de vital importância o diagnóstico precoce e o manejo multidisciplinar envolvendo a equipe cirúrgica, endocrinologistas, radioterapeutas e oncologistas clínicos^2^. O CAT com invasão da artéria carótida comum possui um prognóstico reservado, sendo a remoção cirúrgica em bloco do tumor com ressecção associada da carótida o único tratamento com possibilidade curativa^3^.

Este estudo foi devidamente avaliado e aprovado pelo Comitê de Ética em Pesquisa (CAAE 52619521.2.0000.5411, parecer número 5.086.455).

## PARTE I – SITUAÇÃO CLÍNICA

Uma paciente de 64 anos, do sexo feminino, branca, hipertensa, asmática e independente para atividades básicas de vida diária (ABVD) apresentou queixa de tosse produtiva, febre e dispneia, tendo sido diagnosticada com pneumonia adquirida na comunidade e tratada com antibioticoterapia. Diante da refratariedade dos sintomas após o tratamento convencional, foi solicitada tomografia de tórax, que evidenciou brônquios com paredes espessadas e estrias atelectásicas esparsas, inferindo broncopatia inflamatória. Como achado adicional, observou-se aumento das dimensões do lobo tireoidiano direito, que se projetava inferiormente para o mediastino anterior com desvio da traqueia para a esquerda (Figura 1). Seguiu-se a investigação com ultrassonografia tireoidiana, que evidenciou lobo tireoidiano esquerdo sem alterações (medindo 4,03 × 1,5 × 12 cm e com volume de 4,1 cm^3^) e lobo tireoidiano direito aumentado de tamanho (medindo 6,2 × 3,2 × 3,1 cm e com volume de 30,5 cm^3^), ocupado por nódulo hipoecogênico com ecotextura heterogênea e medindo 5,5 × 3,1 × 2,8 cm. Quanto aos exames laboratoriais, apresentava T4 livre de 0,42 ng/dL, hormônio tireoestimulante (TSH) 1,69 mUI/L e exame de tireoperoxidase (anti-TPO) inferior a 3 UI/mL.

**Figura 1:** Tomografia computadorizada evidenciando: (A) tumor em íntimo contato com porção posterolateral de veia jugular interna direita, parede do esôfago e músculo esternocleidomastoideo direito (corte axial); e (B) mesmo tumor e sua relação com a convergência das veias jugular interna direita e subclávia direita (corte coronal).

Foi realizada punção aspirativa por agulha fina (PAAF) do nódulo tireoidiano, e a análise citológica evidenciou material com alta celularidade, com grupamentos de células com sobreposição, esboço de papila, cariomegalia, cromatina clara e pseudoinclusões citoplasmáticas intranucleares, em meio a material hialino, células gigantes multinucleadas e hemácias. Tais achados são compatíveis com carcinoma papilífero de tireoide (Bethesda VI)^4^.

A paciente foi submetida a tireoidectomia total associada a esvaziamento cervical nível VI^5^, cujo anatomopatológico evidenciou a presença de um carcinoma papilífero de tireoide, variante clássica, com 5 × 4 cm, em lobo direito de tireoide com focos de acometimento em lobo esquerdo e istmo, associado a extensão extratireoideana macroscópica, invasão angiolinfática e perineural, margens cirúrgicas radiais acometidas e metástase em dois de cinco linfonodos ressecados (estadiamento anatomopatológico: pT3bpN1apMx)^6^. Foi classificada como alto risco de recidiva pelos critérios da diretriz da *American Thyroid Association* para câncer diferenciado de tireoide^2^ e submetida a radioiodoterapia, com dose de 150 mCi.

Em seguimento ambulatorial, foram evidenciados sinais de recidiva regional após 9 meses, através de linfonodomegalia de cerca de 6 cm, acometendo níveis cervicais III, IV e V, em íntimo contato com a veia jugular interna direita, parede do esôfago e músculo esternocleidomastoideo direito (MED), incompatível com os níveis de tireoglobulina (Tg) estimulada (TSH = 69 e Tg < 0,04). Diante disso, foi indicado esvaziamento cervical dos níveis II a V, ampliado para esofagectomia cervical.

A paciente foi submetida a nova abordagem cirúrgica. Foi realizada incisão cervical, tipo taco de hóquei, da ponta da mastoide direita até região mediana baixa do pescoço. A dissecção do conglomerado linfonodal (CL) foi iniciada pela cadeia jugulocarotídea inferior (nível IV) e porção inferior do triângulo posterior do pescoço (nível VB). Durante essa etapa, observou-se ausência de plano de clivagem entre o CL e a veia jugular interna direita (VJID) e insinuação do mesmo para próximo da veia subclávia direita (VSD). Optou-se pela ligadura da VJID e claviculotomia para melhor visibilidade e dissecção da VSD. Procedeu-se com esvaziamento dos níveis II, III e V. No nível V, não foi visibilizado plano de clivagem entre o nervo acessório direito e o CL, sendo optado por secção do mesmo. Já no nível II, foi realizada a ligadura da porção proximal da VJID. Durante a dissecção da transição dos níveis III e IV, notou-se que o CL não apresentava plano de clivagem com a artéria carótida comum direita (ACCD), com o MED, além de se estender para o nível VI direito, também não apresentando plano de clivagem com porção proximal do esôfago cervical^5^. Optou-se por secção do MED em sua porção proximal e próximo de sua inserção esternoclavicular e ressecção de um segmento de cerca de 3,5 cm, em parede posterolateral do esôfago, cujo defeito foi prontamente corrigido através de esofagorrafia em dois planos, associada a cobertura com *patch* de musculatura adjacente, além de jejunostomia a Witzel, pela gastrocirurgia. Diante do achado intraoperatório de ausência de plano de clivagem entre o CL e ACCD, foi solicitada avaliação pela equipe da Cirurgia Vascular.

Caso optasse por prosseguir com a ressecção em bloco da carótida comum, as opções de tática cirúrgica vascular seriam:

- Ligadura da artéria carótida comum; ou
- *Shunt* vascular temporário para reconstrução vascular definitiva:
  - Reconstrução vascular com prótese de politetrafluoretileno (PTFE) ou dácron;
  - Reconstrução vascular com veia autóloga; ou
  - Reconstrução vascular com artéria autóloga.

## PARTE II – O QUE FOI FEITO

Diante da ausência de plano clivagem entre o CL e a ACCD, procedeu-se à dissecção e ao isolamento da ACCD, da raiz do pescoço até bifurcação carotídea, e das artérias carótidas externa e interna da bifurcação carotídea até seu cruzamento com o ventre posterior do músculo digástrico. Em seguida, foi feito reparo das mesmas com fita cardíaca.

Optou-se pela utilização de *shunt* vascular temporário e reconstrução vascular definitiva com veia autóloga; procedeu-se com a dissecção e exérese do segmento distal da veia safena magna (VSM).

Após administração de 5.000 UI endovenosa de heparina não fracionada, foi realizado clampeamento proximal na ACCD e distal nas artérias carótidas interna direita (ACID) e externa direita (ACED). Realizou-se a arteriotomia transversa na ACID e na ACCD com distância segura para ressecção oncológica, seguida de implante do *shunt* carotídeo temporário Pruitt F3^®^ (LeMaitre Vascular, Burlington, EUA). Após o controle vascular, foi possível a ressecção do CL, juntamente com os segmentos da ACCD e esofágico acometidos (Figuras 2 e 3).

**Figura 2:** Peça cirúrgica ressecada evidenciando o conglomerado linfonodal e sua relação com segmento da artéria carótida comum direita. À esquerda: lâmina de bisturi número 15, para escala de tamanho.

**Figura 3:** Fotografia intraoperatória do tempo cirúrgico, evidenciando o posicionamento e funcionamento do *shunt* vascular após a remoção do conglomerado linfonodal. O cadarço vascular vermelho repara a artéria carótida comum, enquanto os cadarços azuis reparam as artérias carótidas interna e externa.

Foi realizada interposição da VSM com anastomoses terminoterminais proximal com a ACCD e distal na bifurcação carotídea, com fio de polipropileno 6-0. Ao final do procedimento, foi retirado o *shunt* carotídeo temporário, e observou-se pulsatilidade palpável pré e pós-anastomoses (Figura 4).

**Figura 4:** Fotografia intraoperatória da ponte vascular da carótida direita por interposição da veia safena magna. Nota-se arteriorrafia na carótida interna no local onde foi posicionada a parte distal do *shunt* vascular.

A revisão de lâmina do anatomopatológico da primeira cirurgia evidenciou carcinoma anaplásico da tireoide de variante rabdoide, correspondendo a 10% da neoplasia, evoluindo a partir de carcinoma papilífero da tireoide de variante clássica, correspondendo a 90% da neoplasia, com presença de extensão extratireoideana, invasão angiolinfática e perineural e metástase em dois dos cinco linfonodos acometidos.

Já o anatomopatológico da peça do esvaziamento cervical evidenciou metástase de carcinoma papilífero de tireoide em três linfonodos e de carcinoma anaplásico de tireoide em três dos 16 linfonodos avaliados.

Por fim, a paciente foi submetida a tratamento adjuvante com radioterapia e quimioterapia. No momento, a paciente está em seguimento ambulatorial há cerca de 7 meses do final da adjuvância, sem apresentar sinais de recidiva locorregional.

## DISCUSSÃO

A invasão vascular, compressão vascular ou trombose são complicações raras do câncer diferenciado de tireoide^3^^,^^7^. A estrutura vascular mais comumente afetada com oclusão ou invasão é a veia jugular interna, enquanto a artéria carótida é menos comum de ser afetada^3^^,^^8^.

O fator mais importante em qualquer ressecção em bloco planejada com reconstrução vascular é a avaliação do fluxo cerebral contralateral e do manejo intraoperatório do tempo de isquemia^3^^,^^7^.

É quase consenso entre os especialistas que, no caso de invasão vascular focal, a parede do vaso pode ser ressecada após o controle proximal e distal apropriado, seguido de reconstrução com *patch*^3^.

Em alguns pacientes com câncer de tireoide avançado, baixa expectativa de vida, prognóstico reservado, sangramento de difícil controle com risco de óbito, coto trombosado com risco de embolização durante a manipulação e lesão neurológica grave com sequela prévia, a ressecção da artéria carótida sem reconstrução vascular é uma opção^7^^-^9. Porém, a ressecção seguida de reconstrução é superior à ligadura simples, por evitar maiores complicações neurológicas pós-operatórias^10^.

Assim como em outras situações, após a decisão pela reconstrução vascular e quando a carótida está envolvida, porém não ocluída, o *shunt* torna-se um procedimento indispensável para permitir uma reconstrução segura da artéria carótida^3^^,^^7^^,^^8^^,^^11^.

A reconstrução pode ser feita com enxerto autólogo, sendo a veia safena magna a mais utilizada, ou biomateriais, como o PTFE. Apesar de sua resistência mecânica, segurança e durabilidade, por não ser aderente, o PTFE fornece uma superfície relativamente não trombogênica, com risco aumentado para infecções^10^.

Entre os fatores de risco para infecção, encontram-se a manipulação da mucosa do trato aerodigestivo, a presença de traqueostomia e a manipulação de tecidos previamente irradiados, além da ocorrência de fístulas salivares^7^.

A melhor opção para a reconstrução é o enxerto de veia autóloga, que fornece a patência mais durável e o menor número de complicações trombóticas e infecciosas^3^^,^^7^.

Os procedimentos de reconstrução vascular podem cursar com complicações neurológicas, porém em incidência menor que a ligadura simples^9^. Apesar disso, os riscos elevados de dano neurológico, morte e baixo índice de cura intimidam muitos cirurgiões a não realizar a ressecção da artéria carótida^10^.

Quanto à conduta vascular no caso relatado, não se optou pela ligadura simples da ACCD devido à paciente ser jovem, independente para ABVD e com possibilidade curativa. Optou-se, então, pelo implante do *shunt* temporário devido à decisão de tal tática cirúrgica ter sido tomada previamente à decisão de exérese em bloco do tumor. Quanto à reconstrução vascular, não foi utilizado enxerto protético devido ao risco aumentado de infecção por lesão esofágica associada, sendo realizada a reconstrução com veia autóloga.

Uma das limitações deste estudo é de não ter sido realizado outro exame de imagem no seguimento pós-operatório para avaliar a perviedade do enxerto.

## CONCLUSÃO

Diante da possibilidade de proceder uma cirurgia curativa da tireoide com a ressecção em bloco associada da carótida, o cirurgião vascular deve estar apto para as opções cirúrgicas nesta situação, sendo o *shunt* temporário e a reconstrução vascular com veia autóloga as mais recomendadas.
